# Eating disorders in the structure of depressive states

**DOI:** 10.1192/j.eurpsy.2022.404

**Published:** 2022-09-01

**Authors:** A. Barkhatova, A. Smolnikova, S. Sorokin

**Affiliations:** Mental Health Research Center, Department Of Endogenous Mental Disorders And Affective Conditions, Moscow, Russian Federation

**Keywords:** Eating disorders, Anorexia nervosa, Depression

## Abstract

**Introduction:**

Anorexia nervosa is a disease that occurs mainly in adolescent and young girls and is expressed in a conscious, extremely persistent and purposeful desire to lose weight, often reaching severe cachexia with a possible fatal outcome.

**Objectives:**

Clinical and psychopathological analysis of eating disorders and modeling of clinical and dynamic patterns in terms of their association with depressive disorders, improving the criteria for nosological diagnosis, prognosis and therapy.

**Methods:**

58 patients aged 15 to 25 years who were on outpatient and inpatient observation of the clinic were studied.

**Results:**

It was found that eating disorders are divided into 2 types. The first type is an overvalued eating disorder. In this category, the depressive state developed either during the course of the eating disorder or preceded its development. The second type is delusional eating disorder. In this type, the development of the depressive state did not depend on the eating disorder and proceeded independently of it. At each of these levels, three types of dynamics were identified: narrative type of dynamics (44%), implicit type (25%) and type of selective dissociation (22.4%).

**Conclusions:**

Eating disorders in the structure of depression are heterogeneous and have different degrees of association with depressive symptoms and different variants of the dynamics of their course. The revealed patterns make it possible to formulate a clearer idea of the prognosis of the disease as a whole and to optimize the algorithms for the therapeutic intervention of these conditions.

**Disclosure:**

No significant relationships.

